# Prepubertal-type testicular teratoma with a neuroblastoma component in an infant: a case report

**DOI:** 10.3389/fonc.2026.2000023

**Published:** 2026-10-26

**Authors:** Lili Zhang, Jinyuan Mei, Li Zhang, Ling Liu, Yonghui Yang, Lingyan Wang

**Affiliations:** 1 Department of Pathology, Hebei Children’s Hospital, Hebei Provincial Clinical Research Center for Child Health and Disease, Shijiazhuang, Hebei, China; 2 School of Basic Medical Sciences, Hebei Medical University, Shijiazhuang, Hebei, China

**Keywords:** immature neural tissue, infant, neuroblastoma component, PHOX2B, prepubertal-type testicular teratoma

## Abstract

Prepubertal-type testicular teratomas generally show benign biological behavior and may contain mature or immature neural tissue. A peripheral neuroblastoma component, however, is exceptionally rare and can be difficult to distinguish from conventional immature neuroepithelium. We report a boy aged 4 months and 25 days with a right testicular teratoma treated by testis-sparing excision. Histopathologic examination identified minute, multifocal neuroblastoma components within a prepubertal-type testicular teratoma. The lesional cells showed diffuse strong nuclear expression of paired-like homeobox 2B (PHOX2B). Fluorescence *in situ* hybridization (FISH) showed no MYCN amplification, telomerase reverse transcriptase (TERT) rearrangement, 1p36 deletion, or 11q23 deletion. Imaging revealed no extratesticular primary tumor or metastatic disease, and no recurrence or metastasis was observed during approximately 14 months of follow-up. This case expands the clinicopathologic spectrum of prepubertal-type testicular teratoma and provides additional morphologic, immunophenotypic, and molecular characterization of this rare presentation in infancy.

## 1 Introduction

Prepubertal-type testicular teratoma is among the most common testicular germ cell tumors in children. It belongs to the group of germ cell neoplasia *in situ* (GCNIS)-unrelated germ cell tumors, occurs predominantly in infants and children, and generally follows a benign clinical course (1–3). These tumors may contain mature or immature tissues derived from all three germ layers. Neural and neuroectodermal elements are not uncommon, whereas the development of a relatively discrete neuroblastoma component is exceptionally rare. Teratoma-associated neuroblastoma has been reported mainly in adult postpubertal testicular germ cell tumors. In some cases, the neuroblastoma component constitutes a substantial proportion of the tumor and may give rise to systemic metastases, thereby influencing the clinical course and prognosis (4–6). In contrast, neuroblastoma occurring in pediatric teratomas has rarely been reported, and representative pediatric cases have predominantly involved extratesticular sites (7–10). Because teratomas may themselves contain primitive or immature neural tissue, minute neuroblastic foci can overlap morphologically with conventional immature neural elements, creating a diagnostic challenge. Here, we report a neuroblastoma component occurring within a prepubertal-type testicular teratoma in an infant. By integrating histomorphology, immunophenotype, and molecular genetic findings with comparison of previously reported cases, we highlight the diagnostic basis, major differential diagnoses, and potential clinical significance of this lesion.

## 2 Case report

A boy aged 4 months and 25 days was admitted with a right scrotal mass that had been noticed by his family more than 1 month earlier and had gradually enlarged. He had no fever, scrotal erythema or swelling, significant pain, or urinary symptoms. He was born at term after an uncomplicated perinatal course. There was no history of cryptorchidism or known congenital anomalies and no family history of germ cell tumor or neuroblastoma. Scrotal ultrasonography showed a well-circumscribed mixed cystic and solid lesion in the right testis measuring approximately 32 × 28 × 19 mm **Figure 1**. The solid component showed heterogeneous echogenicity with multiple patchy hyperechoic foci, and color Doppler demonstrated blood flow within the cystic and solid regions. The contralateral testis was unremarkable, and preoperative abdominal ultrasonography showed no definite abnormalities. Testis-sparing tumor excision was performed. Intraoperatively, the lesion was located centrally within the right testis, was well circumscribed, and was completely excised without rupture. The surgical margin was negative, and part of the normal testicular parenchyma was preserved. The resected mass measured 3.0 × 2.5 × 1.8 cm and had an intact capsule. The cut surface was predominantly cystic, with gray-white solid and cartilaginous areas. Microscopically **Figure 2**, tissues derived from all three germ layers, including immature neural tissue, were identified, consistent with a prepubertal-type testicular teratoma. In addition to the immature neural tissue, three relatively discrete hypercellular foci were present, the largest measuring approximately 2.3 mm. The adjacent immature neural tissue measured approximately 4.7 mm in dimension and was morphologically distinct from the compact neuroblastic foci. Of the other two foci, one measured approximately 2.0 mm, whereas the third showed a scattered interstitial distribution and could not be measured reliably. These foci consisted of relatively uniform small round cells arranged in nests, with intervening neuropil-like matrix and focal ganglion cell differentiation; no Schwannian stroma was identified. Immunohistochemically **Figure 3**, the lesional cells showed diffuse strong nuclear positivity for PHOX2B and were positive for synaptophysin (Syn) and chromogranin A (CgA). The Ki-67 proliferation index was approximately 60% in hot spots. The lesional cells were negative for glial fibrillary acidic protein (GFAP), spalt-like transcription factor 4 (SALL4), alpha-fetoprotein (AFP), placental alkaline phosphatase (PLAP), neurofilament protein (NF),and SRY-box transcription factor 2 (SOX2). NF was positive in other neural elements, supporting stain adequacy. No definite yolk sac tumor, embryonal carcinoma, choriocarcinoma, seminoma, or other malignant germ cell tumor component was identified. No lymphovascular invasion was present, and the surgical margin was negative. Scattered neuroblasts were also identified in the interstitium between adjacent seminiferous tubules. Based on the patient’s age, the teratomatous background, the relatively discrete nested neuroblastic proliferation, neuropil-like matrix, focal ganglion cell differentiation, and expression of PHOX2B and neuroendocrine markers, the final diagnosis was prepubertal-type testicular teratoma with a focal PHOX2B-positive neuroblastoma component. To further characterize the molecular genetic features of the neuroblastoma component, FISH was performed on serial sections of formalin-fixed, paraffin-embedded tissue after localization of the target lesion with reference to adjacent immunohistochemically stained sections. No MYCN amplification, TERT rearrangement, 1p36 deletion, or 11q23 deletion was detected. The neuroblastoma component was an unexpected postoperative histologic finding; therefore, urinary vanillylmandelic acid (VMA) and homovanillic acid (HVA) were not measured preoperatively, and postoperative VMA/HVA results were unavailable. To exclude an extratesticular primary peripheral neuroblastic tumor, additional imaging was performed. Abdominal ultrasonography showed no masses in either adrenal gland, the retroperitoneum, or the pelvis, and chest computed tomography (CT) revealed no mediastinal or pulmonary abnormalities. No regional lymph node involvement or distant metastasis was identified on the available examinations. During approximately 14 months of follow-up, which included regular physical examinations and scrotal and abdominal ultrasonography, the patient remained clinically well without local recurrence or metastasis.

**Figure 1:**
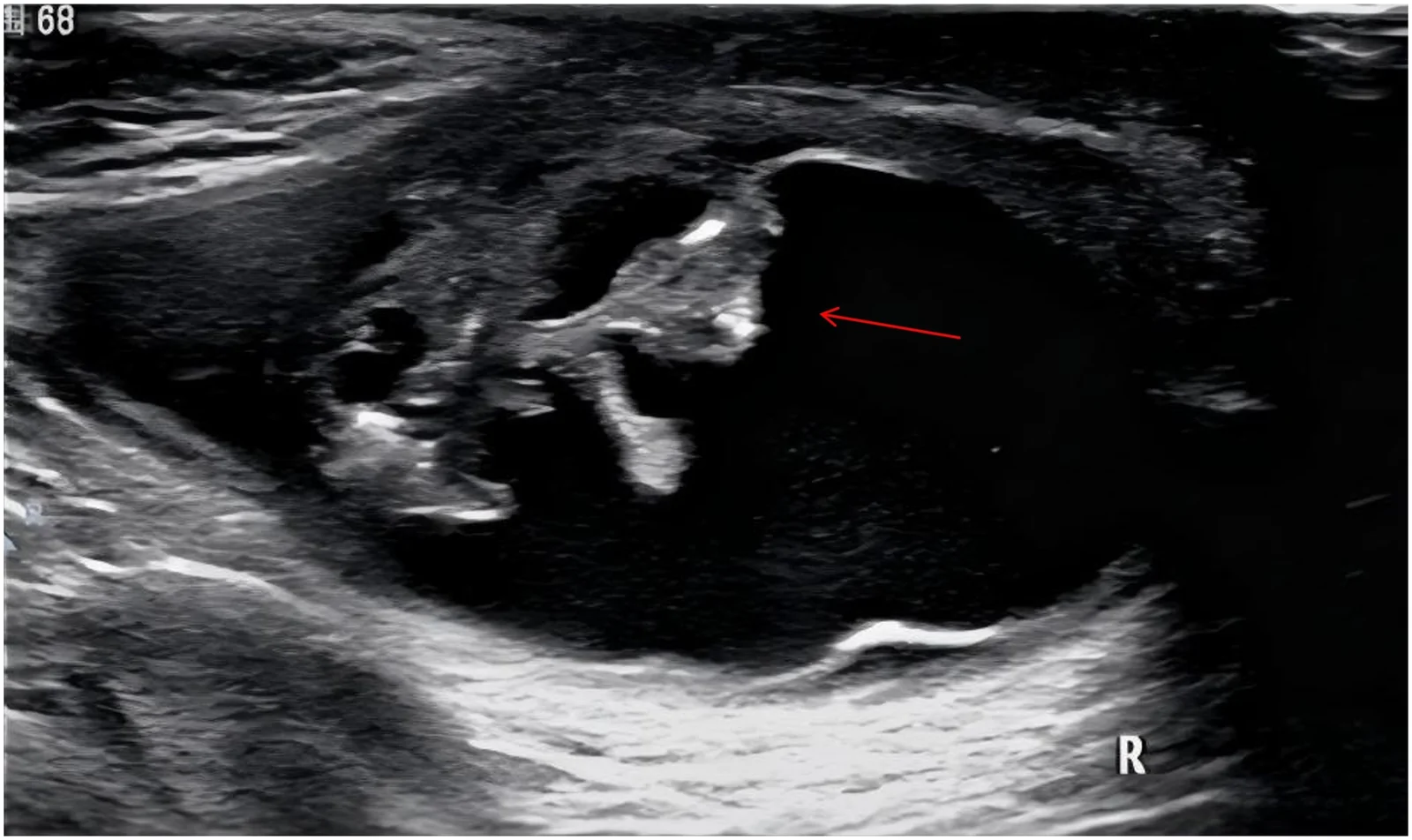
Preoperative scrotal ultrasonography. A well-circumscribed cystic and solid lesion (arrow) was identified within the right testis, measuring approximately 32 × 28 × 19 mm.

**Figure 2:**
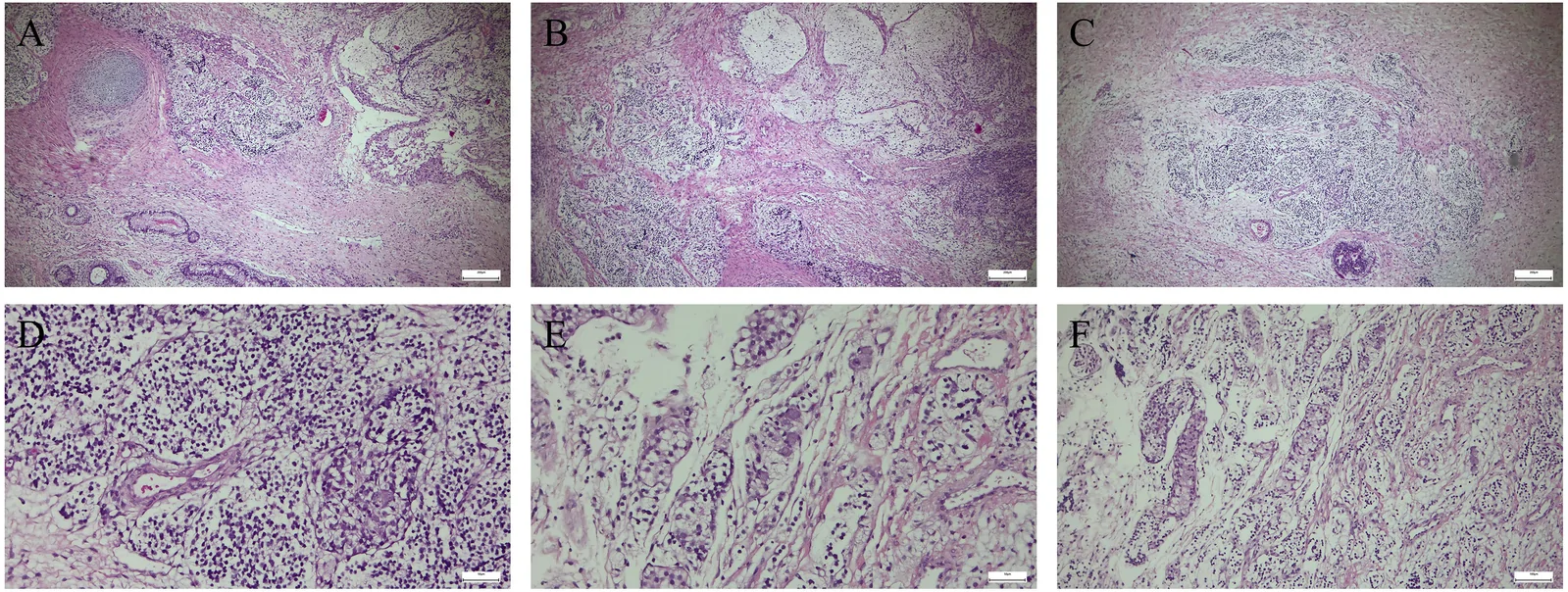
Histologic spectrum of the teratoma and neuroblastic components. Hematoxylin and eosin (H&E) staining. **(A)** Prepubertal-type teratoma containing tissues representing all three germ layers (×50; scale bar = 200μm); **(B)** Nests of neuroblastic foci and adjacent immature neural tissue (×50; scale bar = 200μm); **(C)** The largest discrete neuroblastic focus, measuring 2.3 mm (×50; scale bar = 200μm); **(D)** High-power view of the neuroblastic component showing nests of small round cells and neuropil-like matrix (×200; scale bar = 50μm); **(E)** Focal ganglion-cell differentiation within the neuroblastic component (×200; scale bar = 50μm); **(F)** Scattered neuroblasts in the interstitium between residual seminiferous tubules at the peripheral aspect of the teratomatous lesion (×100; scale bar = 100μm).

**Figure 3:**
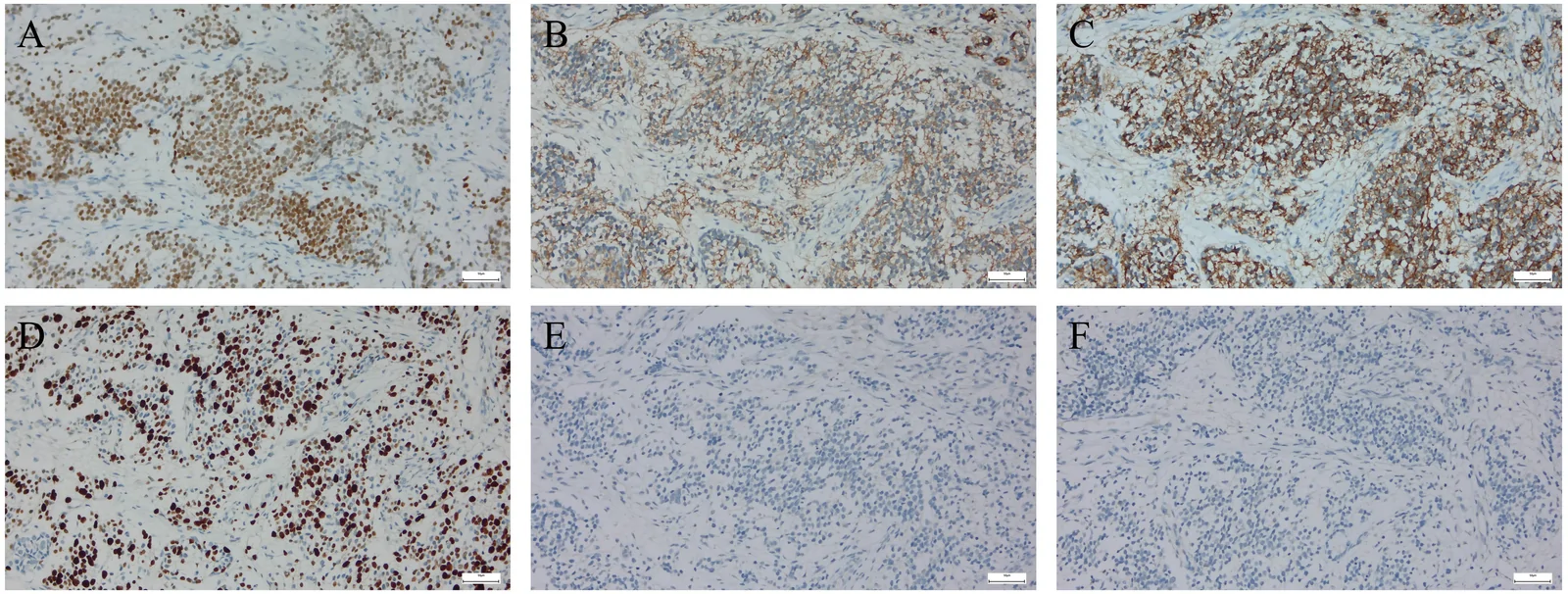
Immunophenotypic features of the microscopic neuroblastic foci. **(A)** Diffuse strong nuclear positivity for PHOX2B; **(B)** cytoplasmic positivity for synaptophysin; **(C)** cytoplasmic positivity for chromogranin A; **(D)** a Ki-67 labeling hotspot with an index of approximately 60%; **(E)** negative staining for GFAP; **(F)** negative staining for SALL4. All panels were examined at ×200 magnification; scale bar = 50 μm.

## 3 Literature review

Published reports indicate that teratomas with a neuroblastoma component are exceptionally rare and have been described in both adults and children, with cases in infants being particularly uncommon **Table 1**. The neuroblastoma component may occur in either mature or immature teratomas and may range from focal or nodular lesions to a component occupying a large proportion of the tumor. Clinical behavior is likewise heterogeneous: localized, completely excised lesions may be associated with prolonged disease-free survival, whereas other cases have shown recurrence, progression, or systemic metastases (4–10).

**Table 1:** Selected published reports of teratoma-associated neuroblastoma or neuroblastic differentiation relevant to the present case.

Study	Age/sex/site	Diagnosis	Immunohistochemistry	Key diagnostic evidence	Metastasis	Treatment outcome
Serrano-Olmo et al., 1993 (4)	Adult male; testis	Mixed germ cell tumor of testis with a prominent neuroblastoma component	NSE positive; ultrastructural examination showed microtubules and rare membrane-limited dense-core granules	Neuroblastoma comprised the majority of the neoplasm; flow cytometry showed a hypertetraploid aneuploid population; cytogenetics showed a hypertriploid modal number of 74 chromosomes	Metastatic/aggressive clinical course reported	Neuroblastoma-directed chemotherapy produced a clinical response; follow-up reported at 2.5 years
Michels et al., 1997 (5)	Adult male; testis	Testicular immature teratoma with neuroblastoma; with intratubular germ cell tumor	Immunohistochemistry supported neuroblastoma	Neuroblastoma morphology; teratomatous background and intratubular germ cell tumor supported germ-cell origin	Multiple systemic metastases	Intensive chemotherapy resulted in partial remission, followed by rapid recurrence and death
Hook et al., 2003 (8)	25-day-old boy; stomach	Grade III gastric immature teratoma with focal neuroblastoma	N/A	Small foci of neuroblastoma within a three-germ-layer immature teratoma	N/A	Complete surgical resection; long-term outcome not reported
Terenziani et al., 2010 (7)	Pediatric; individual sites not specified	Teratoma with a malignant somatic component; neuroblastoma in 3 cases	N/A	Histologically identified neuroblastoma component	N/A	N/A
Ono et al., 2021 (10)	15-year-old girl; ovary	Ovarian mature cystic teratoma with neuroblastoma	Syn, CgA, and TH positive; no MYCN amplification	Neuroblastoma comprised approximately 90%; WES and copy-number analysis showed extensive CN-LOH shared by the neuroblastoma and teratoma, supporting clonal relatedness	Mediastinal and para-aortic lymph-node and bone-marrow metastases	Multimodal therapy; no recurrence 9 years after diagnosis
Felicelli et al., 2023 (9)	13-year-old girl; ovary	Grade 1 ovarian immature teratoma with neuroblastoma	Diffuse PHOX2B positivity	Discrete nodular lesion; neuropil, a maturation spectrum of neuroblasts, and Schwannian stroma; coexisting immature neuroepithelium	N/A	Ovary-sparing cystectomy; long-term outcome not reported
Ziade et al., 2026 (6)	25-year-old man; testis	Mixed nonseminomatous germ cell tumor (teratoma and embryonal carcinoma) with neuroblastoma-like somatic-type malignancy	CD56 and NSE diffusely positive; Syn weak or heterogeneous; S100 focal; PHOX2B not tested	Neuroblastoma-like morphology and neuronal immunophenotype; lesion located within the teratomatous component	No metastasis on initial imaging	Radical orchiectomy plus four cycles of chemotherapy; complete remission
Present case	Boy aged 4 months and 25 days; testis	Prepubertal-type testicular teratoma with multifocal microscopic neuroblastic foci favoring peripheral neuroblastic differentiation	Diffuse strong nuclear PHOX2B positivity; Syn and CgA positive; GFAP, SALL4, AFP, PLAP, and NF negative; Ki-67 hot spot approximately 60%	Three minute foci, largest approximately 2.3 mm; nested small round cells, neuropil-like matrix, and focal ganglion cell differentiation; FISH showed no MYCN amplification, TERT rearrangement, 1p36 deletion, or 11q23 deletion	No extratesticular primary lesion or metastasis identified on available imaging; MIBG and bone-marrow evaluation not performed	Testis-sparing tumor excision; active surveillance; no recurrence or metastasis at approximately 14 months

The table summarizes selected cases with clinicopathologic features directly relevant to the present case and is not intended to represent an exhaustive compilation of all germ cell tumor-associated embryonic neuroectodermal tumors. Cases are listed in chronological order of publication. AFP, alpha-fetoprotein; CgA, chromogranin A; CN-LOH, copy-neutral loss of heterozygosity; FISH, fluorescence *in situ* hybridization; GFAP, glial fibrillary acidic protein; MKI, mitosis-karyorrhexis index; N/A, information not available; NF, neurofilament protein; NSE, neuron-specific enolase; PHOX2B, paired-like homeobox 2B; PLAP, placental alkaline phosphatase; PNET, primitive neuroectodermal tumor; SALL4, spalt-like transcription factor 4; Syn, synaptophysin; TERT, telomerase reverse transcriptase; TH, tyrosine hydroxylase; WES, whole-exome sequencing.

The present case is distinctive because it occurred in an infantile prepubertal-type testicular teratoma and the neuroblastoma component was limited to minute, multifocal foci. Previously reported testicular cases have predominantly involved adult postpubertal or mixed germ cell tumors. This case therefore expands the clinicopathologic spectrum of prepubertal-type testicular teratomas containing a neuroblastoma component.

## 4 Discussion

Prepubertal-type testicular teratoma is a GCNIS-unrelated germ cell tumor that generally behaves benignly in children. In contrast, “teratoma with somatic-type malignancy” is classified within the GCNIS-related germ cell tumor framework; its diagnosis requires an unequivocally expansile somatic-type malignant component and uses an approximate size threshold of 5 mm. The current fifth edition of the WHO classification does not, however, define a separate category or specific diagnostic criteria for a neuroblastoma component occurring within a prepubertal-type testicular teratoma (1, 11, 12). The minute, multifocal neuroblastic foci in this infant therefore illustrate a diagnostic scenario that is not specifically addressed in the current WHO classification. Histologically, the foci showed relatively discrete nested/nodular growth, neuropil-like fibrillary matrix, and focal ganglion cell differentiation, spanning a spectrum from neuroblasts to more mature neuron-like cells. The lesional cells showed diffuse strong nuclear PHOX2B expression and coexpressed Syn and CgA. PHOX2B is an important marker of peripheral neuroblastic differentiation and has high sensitivity and specificity in the differential diagnosis of pediatric small round blue cell tumors (13, 14).

The first major differential diagnosis is conventional immature neural tissue within a teratoma. Immature neuroepithelium typically forms primitive neuroepithelial tubules, rosettes, or sheets and may merge with other mature or immature neural/glial elements. Neural and glial components of immature teratomas may express markers such as GFAP (15). In the present case, the target foci were GFAP-negative, whereas conventional glial and immature neural tissues were present in the adjacent teratoma. This spatial and immunophenotypic distinction argues against interpreting the target foci as merely glial/central-type immature neural tissue. Nevertheless, GFAP negativity alone does not exclude immature neuroepithelium; the relatively discrete nodular growth, neuropil-like matrix, and ganglion cell differentiation are more diagnostically informative.Although the neuroblastic foci were negative for neurofilament protein (NF), adjacent neural elements showed positive staining, supporting the technical adequacy of the stain. This discordant finding should be interpreted cautiously and does not, by itself, exclude peripheral neuroblastic differentiation. The diagnosis therefore relies on the integrated morphologic and immunophenotypic findings rather than on any single marker. The second major differential diagnosis is embryonic-type neuroectodermal tumor (ENET) and other germ cell tumor-associated neuroectodermal lesions. Historical series of germ cell tumor-associated primitive neuroectodermal tumors (PNETs) provide important context for this differential diagnosis. Michael et al. reported 29 young men with germ cell tumor-associated PNETs, with variable patterns of testicular and extratesticular involvement (16). Ulbright et al. subsequently described a neuroblastic tumor with abundant neuropil and true rosettes among 14 PNETs from patients with testicular germ cell tumors (17). These studies demonstrate the morphologic heterogeneity of historical PNET classifications but do not establish neuroblastoma as a formal subtype of ENET under current WHO terminology (1). Moreover, these adult cohorts are not directly comparable to the present case, which occurred in an infant with a prepubertal-type testicular teratoma and showed only minute, multifocal neuroblastic foci. More recent data indicate variable expression of traditional neural markers and greater sensitivity of SOX2 in ENT (ENET; termed ENT in the cited study) (18). Although PHOX2B positivity, neuropil, and ganglion-cell differentiation do not absolutely exclude ENET, the combination of SOX2 negativity, the minute multifocal foci, prepubertal-type teratomatous background, very young age, and absence of progression during approximately 14 months favor peripheral neuroblastic differentiation. A microscopic yolk sac tumor component was also considered. No morphologic component diagnostic of yolk sac tumor was identified in the examined sections, and the target foci were negative for AFP and SALL4 by immunohistochemistry. The preoperative serum AFP level was 48.63 ng/mL at 4 months and 25 days of age, which was within the age-appropriate pediatric reference interval and therefore was not considered elevated (19). During approximately 14 months of follow-up, the serum AFP level decreased to 2.95 ng/mL. These serum AFP findings provided no biochemical evidence of AFP elevation; however, a normal age-adjusted serum AFP level alone cannot exclude a microscopic yolk sac tumor component. Therefore, the exclusion of yolk sac tumor in this case was based primarily on the absence of diagnostic yolk sac tumor morphology together with negative AFP and SALL4 immunohistochemistry. Metastatic neuroblastoma to the testis from an extratesticular primary site must also be excluded. If the neuroblastoma component originated from a primary tumor in the adrenal gland, retroperitoneum, or another site of the sympathetic nervous system, the diagnostic, staging, and therapeutic pathways would differ substantially. Postoperative imaging of both adrenal glands, the retroperitoneum, pelvis, mediastinum, and lungs revealed no primary lesion or metastasis. Moreover, the neuroblastic foci were located within the teratoma and showed focal continuity with adjacent immature neural tissue, favoring a teratoma-associated origin over metastatic disease. The absence of VMA/HVA data, because the lesion was an unexpected postoperative finding, remains a limitation of the clinical exclusion process. The focal continuity between the neuroblastic foci and adjacent immature neural tissue is noteworthy. The ovarian case reported by Felicelli et al. similarly showed a discrete neuroblastoma nodule coexisting with immature neuroepithelium (9), suggesting that neural elements within a teratoma may acquire a more autonomous peripheral neuroblastic growth pattern during development. However, morphologic continuity alone cannot establish clonal evolution or true malignant transformation. If feasible, comparative molecular analysis of microdissected components would be more informative than additional immunohistochemistry for determining whether the two components are clonally related. In the absence of such paired molecular evidence, this relationship should be regarded as a developmental hypothesis rather than an established mechanism. Pathologic grading and prognostic interpretation should also be approached cautiously. The International Neuroblastoma Pathology Classification (INPC) primarily evaluates primary peripheral neuroblastic tumors on the basis of Schwannian stroma, degree of neuroblastic differentiation, mitosis-karyorrhexis index (MKI), and age (20, 21). In the present case, Schwannian stroma was absent, MKI was assessed in 5,000 evaluable neuroblastic cells and was <2%, and focal ganglion cell differentiation was present. Although the Ki-67 index was approximately 60% in hot spots, Ki-67 is not a core parameter of INPC grading.The Ki-67 labeling index and the mitosis-karyorrhexis index (MKI) assess different aspects of cellular proliferation and are not interchangeable,We avoid using any single indicator alone to infer the biological behavior of the lesion. More importantly, because the lesion occurred in the special context of a teratoma and had an extremely low overall tumor burden, conventional risk-stratification systems for primary neuroblastoma should not be directly applied for pathologic grading or prognostic interpretation. Molecular genetic analysis further characterized the biological features of the present case. MYCN amplification is a well-established adverse prognostic factor in neuroblastoma and has been incorporated into the International Neuroblastoma Risk Group (INRG) risk-classification system (22). Deletions of 1p36 and 11q23 are associated with disease progression and poorer survival outcomes (23, 24). TERT rearrangements can activate telomerase and define a subgroup of neuroblastomas with aggressive biological behavior and unfavorable prognosis (25, 26). In the present case, FISH detected no MYCN amplification, TERT rearrangement, 1p36 deletion, or 11q23 deletion, indicating the absence of these recognized adverse molecular genetic features. This finding is consistent with the minute and localized nature of the lesions and the absence of recurrence or metastasis during approximately 14 months of postoperative follow-up. However, these molecular risk markers were established primarily in conventional primary neuroblastoma, and their prognostic value in minute teratoma-associated neuroblastic lesions has not been validated. Therefore, they should not be used to infer the long-term prognosis of this patient (27). From a clinical-management perspective, outcomes in previously reported cases have varied widely. Some adult cases developed systemic metastases and required neuroblastoma-directed systemic therapy (4, 5), whereas localized and completely resectable lesions have also been associated with favorable outcomes (6, 9, 10). In the present case, Scattered neuroblasts appeared in the stroma between adjacent convoluted tubules, which were found to be associated with teratoma lesions. No lymphovascular invasion was seen, and the surgical margins were negative.In addition, no extratesticular primary neuroblastoma or metastatic disease was identified on the available imaging, and no recurrence or progression was observed during approximately 14 months of follow-up. Taken together, these findings favor local intratesticular extension or infiltration of the teratoma-associated neuroblastic component rather than an independent metastatic deposit. Postoperative active surveillance was therefore considered reasonable. This approach, however, should not be extrapolated to larger, expansile, or metastatic lesions. Given the current follow-up of approximately 14 months, continued long-term clinical, imaging, and tumor-marker surveillance is warranted. This report has several limitations. First, because the neuroblastoma component was an unexpected postoperative finding, VMA/HVA results were unavailable. Second, the existing literature consists largely of single case reports or small series; terminology in some earlier reports does not fully correspond to the current WHO classification, and immunohistochemical and molecular data are limited, restricting direct comparison across cases. Moreover,because metaiodobenzylguanidine (MIBG) scintigraphy and bone marrow evaluation were not performed, occult systemic neuroblastoma cannot be completely excluded. Finally, the current follow-up of approximately 14 months remains relatively short, and long-term surveillance is required to clarify the biological behavior of this lesion.

## 5 Conclusion

We report a rare case of prepubertal-type testicular teratoma with minute, multifocal neuroblastic foci in an infant aged 4 months and 25 days. The integrated clinicopathologic findings favor a peripheral neuroblastic component within the teratoma, while the biological significance of such microscopic lesions remains uncertain. Given the rarity of this finding in infants, careful pathologic evaluation and longer clinical follow-up are warranted to better define its clinical behavior.

## Data Availability

The original contributions presented in the study are included in the article/supplementary material. Further inquiries can be directed to the corresponding authors.
